# Keratoprosthesis Surgery: Evolution and Global Adaptations

**DOI:** 10.18502/jovr.v19i3.17132

**Published:** 2024-09-16

**Authors:** Mohammad Soleimani, Ali R. Djalilian

**Affiliations:** ^1^Department of Ophthalmology and Visual Sciences, University of Illinois at Chicago, Chicago, IL, USA; ^3^Ali R. Djalilian: https://orcid.org/0000-0002-1489-0724; ^4^Mohammad Soleimani: https://orcid.org/0000-0002-6546-3546

Keratoprosthesis (KPro) surgery, specifically the Boston Type 1, has emerged as a pivotal solution for patients with bilateral end-stage corneal blindness, where traditional grafting methods have failed. This artificial cornea has broadened the scope of possibilities for restoring vision, especially in patients with severe ocular surface diseases. However, accessibility and costs can limit its application, particularly in lower-resourced countries.

The interest in the Boston keratoprosthesis worldwide over the past decade can be attributed to significant modifications by Prof. Dohlman and colleagues at the Massachusetts Eye and Ear Infirmary. These modifications have led to a drastic drop in infection rates, thanks in part to the use of lifelong vancomycin drops and large diameter bandage contact lenses changed at regular intervals. Moreover, the Boston Type 1 keratoprosthesis has been made more accessible in developing countries through cost reduction to a fifth of the original price for the device from Boston. In the same direction, we have seen the development of auroKPro based on the Boston Keratoprosthesis in India, with encouraging outcomes that are affordable in populations with low economic resources in developing countries.^[1]^

A recent study from Tehran, Iran, detailing the adaptation of the Boston Type 1 KPro into a locally produced variant named ORC-KPro, marks another significant step in making this technology more widely accessible.^[2]^ The study's findings on the ORC-KPro's short-term anatomical and visual outcomes are promising, showing considerable success in patient recovery with minimal complications, including effective management of retroprosthetic membrane (RPM) formation.^[3]^ This KPro demonstrates that local adaptations of complex medical technologies can help meet the requirements of specialized medical treatments under certain circumstances.

In summary, these studies underscore the importance of ongoing research and development in the field of ocular prosthetics, particularly as a viable option in settings where traditional KPro may not be accessible or affordable. We encourage the authors to continue their research to assess long-term outcomes of this modified Kpro.
